# Supplementary material for: Iterative improvement in the automatic modular design of robot swarms
Source: PeerJ Comput Sci. 2020 Dec 7;6:e322. doi: 10.7717/peerj-cs.322 (PMC7924708; doi:10.7717/peerj-cs.322)
Supplement: Supplemental Information 3 [file peerj-cs-06-322-s003.zip › argos3/doc/api/standalone/a00486_source.html]

ARGoS: plugins/robots/generic/control\_interface/ci\_altitude\_sensor.cpp Source File


- Main Page
- Related Pages
- Namespaces
- Classes
- Files

- File List
- File Members

# plugins/robots/generic/control\_interface/ci\_altitude\_sensor.cpp

Go to the documentation of this file.

```
00001 
00007 #include "ci_altitude_sensor.h"
00008 
00009 #ifdef ARGOS_WITH_LUA
00010 #include <argos3/core/wrappers/lua/lua_utility.h>
00011 #endif
00012 
00013 namespace argos {
00014 
00015   /****************************************/
00016   /****************************************/
00017 
00018 #ifdef ARGOS_WITH_LUA
00019   void CCI_AltitudeSensor::CreateLuaState(lua_State* pt_lua_state) {
00020     CLuaUtility::AddToTable(pt_lua_state, "altitude", m_fReading);
00021   }
00022 #endif
00023 
00024   /****************************************/
00025   /****************************************/
00026 
00027 #ifdef ARGOS_WITH_LUA
00028   void CCI_AltitudeSensor::ReadingsToLuaState(lua_State* pt_lua_state) {
00029     lua_pushnumber(pt_lua_state, m_fReading);
00030     lua_setfield(pt_lua_state, -2, "altitude");
00031   }
00032 #endif
00033 
00034 
00035   /****************************************/
00036   /****************************************/
00037 
00038   Real CCI_AltitudeSensor::GetReading() const {
00039     return m_fReading;
00040   }
00041 
00042 }
```

---

Generated on 10 Jul 2018 for ARGoS by 
 1.6.1
